# Supplementary material for: Anti-fibrotic activity of a rho-kinase inhibitor restores outflow function and intraocular pressure homeostasis
Source: eLife. 2021 Mar 30;10:e60831. doi: 10.7554/eLife.60831 (PMC8009676; doi:10.7554/eLife.60831)
Supplement: Supplementary file 1. [file elife-60831-supp1.docx]

Supplementary Table 1:

Estimated pressures within SC lumen as a function of clamped IOP levels using two-series resistor model of conventional outflow pathway described previously**.**


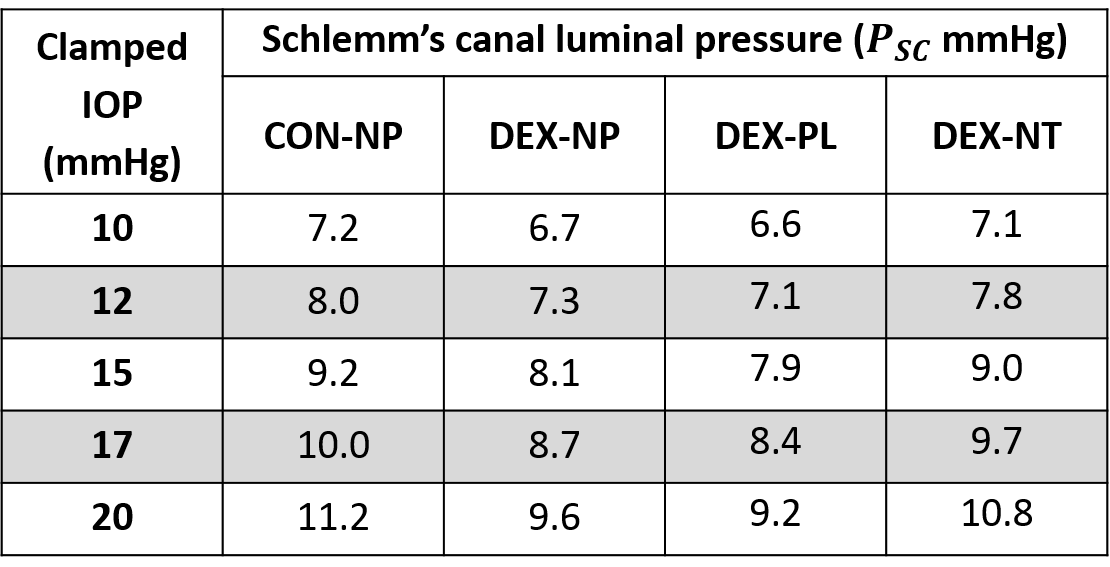


IOP: intraocular pressure, PL: placebo, NT: netarsudil, DEX: dexamethasone
