# Supplementary material for: Anti-fibrotic activity of a rho-kinase inhibitor restores outflow function and intraocular pressure homeostasis
Source: eLife. 2021 Mar 30;10:e60831. doi: 10.7554/eLife.60831 (PMC8009676; doi:10.7554/eLife.60831)
Supplement: Transparent reporting form [file elife-60831-transrepform.pdf]

eLIFE

Westbrook Centre, Milton Road
Cambridge CB4 1YG
UK

P 01223 855340
W elifesciences.org
T @elife

eLife's transparent reporting form

We encourage authors to provide detailed information *within their submission* to facilitate the interpretation and replication of experiments. Authors can upload supporting documentation to indicate the use of appropriate reporting guidelines for health-related research (see EQUATOR Network), life science research (see the BioSharing Information Resource), or the ARRIVE guidelines for reporting work involving animal research. Where applicable, authors should refer to any relevant reporting standards documents in this form.

If you have any questions, please consult our Journal Policies and/or contact us: editorial@elifesciences.org.

Sample-size estimation

- You should state whether an appropriate sample size was computed when the study was being designed
- You should state the statistical method of sample size computation and any required assumptions
- If no explicit power analysis was used, you should describe how you decided what sample (replicate) size (number) to use

Please outline where this information can be found within the submission (e.g., sections or figure legends), or explain why this information doesn't apply to your submission:

For electron microscopy scoring and IOP measurements we performed power analysis to determine cohort sizes. For effect size of dexamethasone and netarsudil on IOPs, we used data from two previous studies (PMIDs: 30651311, 27085895) Since IOPs are normally distributed, we used a two-tailed t-test with alpha at 0.05, unequal variance then inflated by 5% for the Wilcoxon rank sum test to achieve 80 and 90% power.

Replicates

- You should report how often each experiment was performed
- You should include a definition of biological versus technical replication
- The data obtained should be provided and sufficient information should be provided to indicate the number of independent biological and/or technical replicates
- If you encountered any outliers, you should describe how these were handled
- Criteria for exclusion/inclusion of data should be clearly stated
- High-throughput sequence data should be uploaded before submission, with a private link for reviewers provided (these are available from both GEO and ArrayExpress)

Please outline where this information can be found within the submission (e.g., sections or figure legends), or explain why this information doesn't apply to your submission:

Patient inclusion and IOP outlier criteria are outlined. Outflow facility pressure step outlier criteria are described according to established criteria (PMID: 26949939). Technical replicates for single IOP readings in mice and AFM replicate measurements are described. Sampling for histology are indicated . Sampling for TEM quantification of BMMs are indicated and based upon published precident (PMID:25028360) Biological replicates are clearly indicated in figure legends as "n" numbers.

eLife Sciences Publications, Ltd is a limited liability non-profit non-stock corporation incorporated in the State of Delaware, USA, with company number 5030732, and is registered in the UK with company number FC030576 and branch number BR015634 at the address Westbrook Centre, Milton Road Cambridge CB4 1YG, UK | March 2019

Statistical reporting

- Statistical analysis methods should be described and justified
- Raw data should be presented in figures whenever informative to do so (typically when N per group is less than 10)
- For each experiment, you should identify the statistical tests used, exact values of N, definitions of center, methods of multiple test correction, and dispersion and precision measures (e.g., mean, median, SD, SEM, confidence intervals; and, for the major substantive results, a measure of effect size (e.g., Pearson's r, Cohen's d)
- Report exact p-values wherever possible alongside the summary statistics and 95% confidence intervals. These should be reported for all key questions and not only when the p-value is less than 0.05.

Please outline where this information can be found within the submission (e.g., sections or figure legends), or explain why this information doesn't apply to your submission:

All raw data points are shown in figures. Summary of statistical methods used and justification are described in detail in Methods. Statistical tests employed for quantification of immunofluorescence intensity, semi-quantitative analysis of JCT ECM and quantification of BMM were determined by independent statistician (DR. Sandra Stinnett). P values and 95% confidence intervals are shown where appropriate alongside data in Results and in figure legends.

(For large datasets, or papers with a very large number of statistical tests, you may upload a single table file with tests, Ns, etc., with reference to sections in the manuscript.)

Group allocation

- Indicate how samples were allocated into experimental groups (in the case of clinical studies, please specify allocation to treatment method); if randomization was used, please also state if restricted randomization was applied
- Indicate if masking was used during group allocation, data collection and/or data analysis

Please outline where this information can be found within the submission (e.g., sections or figure legends), or explain why this information doesn't apply to your submission:

Masked and randomized drug treatment of mice are described. Masked and randomized outflow facility measurements are indicated. Masked marking and quantification of immunofluorescence images are detailed. Masked marking and grading of transmission electron microscopy images are detailed. Interobserver and intraobserver reproducibility of semi-automatic segmentation are outlined.

Additional data files ("source data")

- We encourage you to upload relevant additional data files, such as numerical data that are represented as a graph in a figure, or as a summary table
- Where provided, these should be in the most useful format, and they can be uploaded as "Source data" files linked to a main figure or table
- Include model definition files including the full list of parameters used
- Include code used for data analysis (e.g., R, MatLab)
- Avoid stating that data files are "available upon request"

Please indicate the figures or tables for which source data files have been provided:

Source data for Figure 1 is located in Table 1. Source data for all other figures is provided in individually labeled excel files. For example, "Figure 2-Source data 1".
